# Diagnostic Performance of Artificial Intelligence in Detecting COVID-19 Pneumonia on Chest Imaging

**DOI:** 10.7759/cureus.101775

**Published:** 2026-01-18

**Authors:** Savannah R Chapman, Lauren Willner, Alex Abouafech, Christian Roberti, Courtney Willner

**Affiliations:** 1 School of Medicine, Lake Erie College of Osteopathic Medicine, Bradenton, USA; 2 Department of Emergency Medicine, Lake Erie College of Osteopathic Medicine, Bradenton, USA

**Keywords:** artificial intelligence in radiology, chest x-ray (cxr), covid-19, covid-19 pneumonia, ct (computed tomography) imaging, deep learning artificial intelligence, diagnostic performance, thoracic radiology

## Abstract

The COVID-19 pandemic highlighted the need for rapid, accurate, and accessible diagnostic tools. Chest imaging modalities, including chest radiography (CXR) and computed tomography (CT), provided valuable diagnostic information and prompted the development of artificial intelligence (AI) systems to support image interpretation and improve workflow efficiency. This literature review synthesizes current evidence on the diagnostic performance, limitations, and clinical implications of AI models in COVID-19 pneumonia detection through CXR and CT evaluation. A PubMed search was conducted through October 2025 to identify studies evaluating AI systems for the detection of COVID-19 pneumonia using CXR and CT. Studies reporting diagnostic performance metrics, including sensitivity, specificity, accuracy, or area under the curve (AUC), were included. Study quality and risk of bias were assessed using the Quality Assessment of Diagnostic Accuracy Studies-2 (QUADAS-2) tool. Eleven studies met the inclusion criteria. CXR-based AI systems demonstrated sensitivities from 80% to 98% and specificities from 82% to 96%, often comparable to radiologist performance. CT-based AI models achieved accuracies between 90% and 96%. AI models demonstrated strong internal diagnostic performance on CXR and CT but showed reduced accuracy with external validation, underscoring limitations related to generalizability and retrospective study designs. AI models demonstrate promising diagnostic performance for detecting COVID-19 pneumonia on chest imaging and may enhance radiologist efficiency. However, challenges related to generalizability, model adaptability, and clinician trust remain. Future research should prioritize external validation and transparent reporting to ensure the safe and effective integration of AI into clinical practice.

## Introduction and background

The COVID-19 pandemic created an urgent need for rapid and accurate diagnostic strategies. Although reverse transcription polymerase chain reaction (RT-PCR) remains the gold standard for confirming SARS-CoV-2 infection, it does not provide information regarding pulmonary involvement and has limitations related to sensitivity, turnaround time, and accessibility in many settings [1]. Imaging modalities such as chest radiography (CXR) and computed tomography (CT) offer rapid, widely available, and inexpensive means of assessing lung pathology, making them valuable tools for the identification and monitoring of COVID-19 pneumonia [2].

The increased demand for imaging interpretation has accelerated the development of AI-based diagnostic tools. Artificial intelligence (AI) algorithms have demonstrated strong performance in medical image classification, which may assist in diagnosis. However, model performance can be influenced by dataset quality, disease prevalence, and generalizability across different clinical environments [1]. This review aims to summarize studies evaluating the diagnostic performance of AI models in detecting COVID-19 pneumonia on CXR and CT, with emphasis on performance metrics, limitations, and potential clinical impact.

## Review

Methods

Literature Search

A literature search was conducted in PubMed (National Library of Medicine) through October 2025 using the following search terms: "artificial intelligence AND COVID-19 pneumonia AND CXR” and “artificial intelligence AND COVID-19 pneumonia AND CT.” The search was limited to English-language, full-text studies published between January 2020 and October 2025. Reference lists of included studies were also screened to identify additional relevant articles. 

Studies were considered eligible if they evaluated AI-based models for the detection or diagnosis of COVID-19 pneumonia on chest imaging (CXR or CT) and reported diagnostic performance metrics, including sensitivity, specificity, accuracy, or area under the curve (AUC). Editorials, reviews, letters, case reports, animal studies, and non-English publications were excluded. Although this review was not preregistered, inclusion and exclusion criteria were applied consistently to enhance transparency and minimize selection bias.

**Figure 1:**
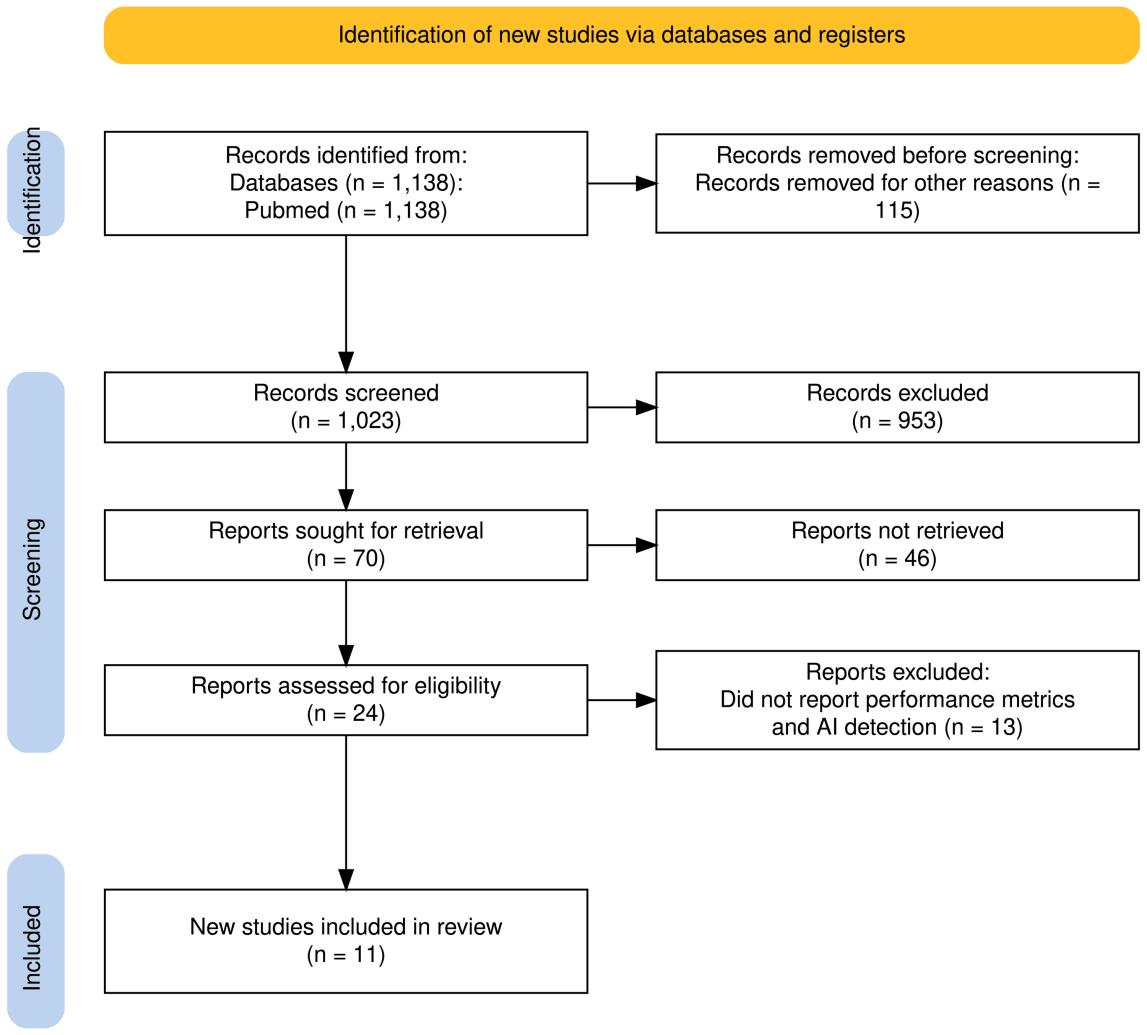
PRISMA flow diagram summary of methodology n: number; AI: artificial intelligence; PRISMA: Preferred Reporting Items for Systematic Reviews and Meta-Analyses

Titles and abstracts identified through database searches were screened for relevance. Full-text articles were reviewed when eligibility was uncertain. In total, 11 studies were included for qualitative synthesis. Although this was not a systematic review, the study selection process followed the general framework of the Preferred Reporting Items for Systematic Reviews and Meta-Analyses (PRISMA) 2020 statement, without a preregistered protocol [3]. The PRISMA methodology is illustrated in Figure 1, summarizing the number of studies identified, screened, retrieved, excluded, and included. 

Quality Assessment 

A quality assessment tool that evaluated diagnostic accuracy studies, Quality Assessment of Diagnostic Accuracy Studies-2 (QUADAS-2), was utilized to examine potential biases across four key domains [4]. The four key areas include patient selection methods, index testing, reference standard, and study flow and timing, as well as their assessment in applicability.

Review

Overview of AI

AI refers to computer systems that are designed to perform tasks that typically require human intelligence. This includes machine learning techniques such as DL with CNNs, which can automatically learn spatial and textural features. CNNs are particularly well suited in medical imaging as they can detect subtle visual patterns [5,6]. 

AI in Imaging Before COVID-19

AI has been studied in radiology since the 1960s, with advances following the integration of artificial neural networks and AI-based computer-aided detection (CAD) software [5]. Prior to the COVID-19 pandemic, AI applications in chest imaging were already established in chest imaging for identifying various lung pathologies, including tuberculosis, lung nodules, and cancer [7,8]. With the emergence of the COVID-19 pandemic, these established methods were rapidly adapted and optimized for the detection of COVID-19 pneumonia [9]. 

COVID-19 Imaging Findings on CXR and CT

**Figure 2:**
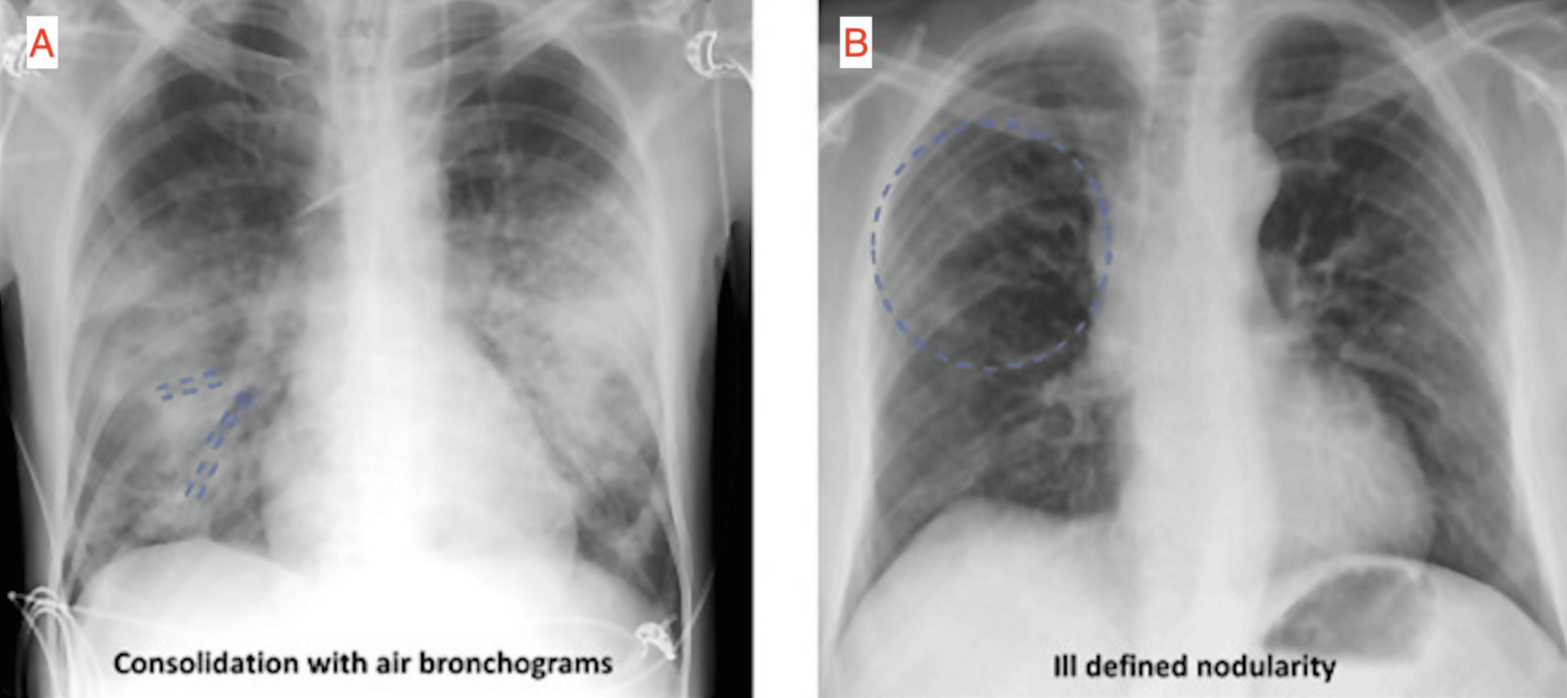
Representative CXR images of COVID-19 pneumonia CXR: chest x-ray; AP: anteroposterior (A) AP CXR shows consolidation with air bronchograms in the right lung. (B) AP CXR shows ill-defined nodularity of the right lung Image adapted and reproduced from Bahra et al. [10] under the CC BY 4.0 license

**Figure 3:**
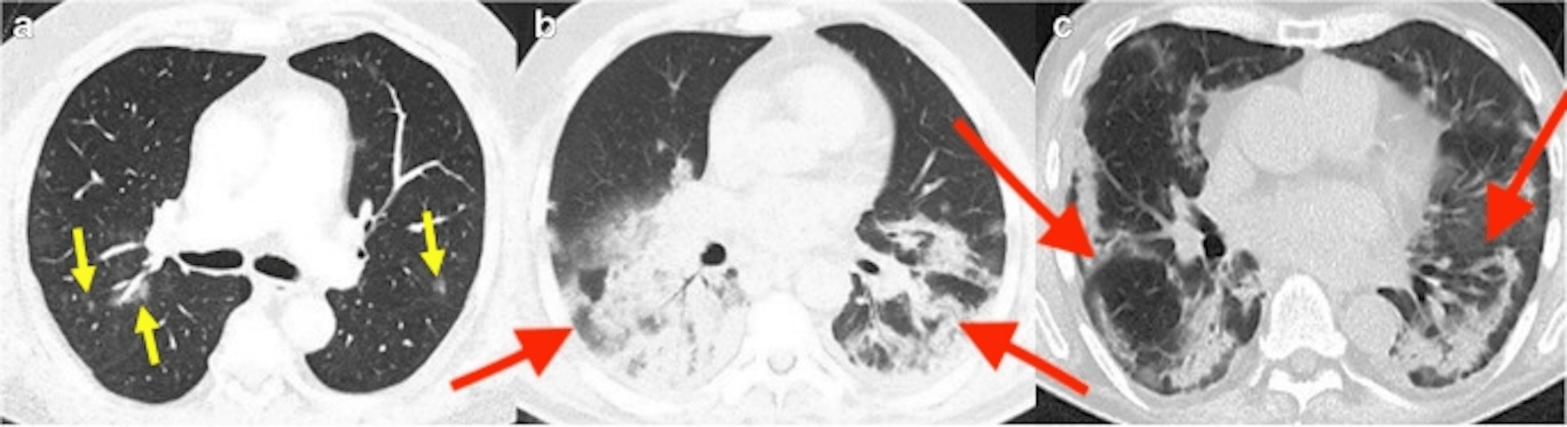
Representative CT findings in COVID-19 pneumonia CT: computed tomography Transverse CT images show (A) ill-defined ground-glass nodules bilaterally (yellow arrows), (B) extensive ill-defined consolidations with positive air bronchograms in bilateral lower lobes (red arrows), and (C) consolidations in the lung periphery sparing the subpleural space (red arrows). Image adapted and reproduced from Afshar-Oromieh et al. [2] under the CC BY 4.0 license

To better understand these adaptations, it is essential to review the characteristic imaging features of COVID-19 pneumonia on CXR and CT, as illustrated in Figures 2A-2B and Figures 3A-3C, respectively. Common findings on CXR include bilateral air-space opacities, including consolidation, ground-glass opacities, and reticular abnormalities with peripheral predominance [2]. Additional findings include consolidation with air bronchograms and ill-defined nodularity (Figures 2A-2B) [10]. These findings are nonspecific but supportive in an appropriate clinical context. Typical CT findings include peripheral ground-glass opacities, fine reticular opacities, and vascular thickening [2,11]. Figures 3A-3C demonstrate COVID-19 pneumonia findings on CT [2].

Summary of Included Studies

After applying our search strategy, a total of 11 studies were included in this review. Table 1 provides a summary of all included studies evaluating AI performance in the detection of COVID-19 across CXR and CT modalities. Most studies included retrospective cohort designs and a spectrum of DL models and other transfer learning modalities. The key findings of these studies in relation to our review are further presented in the discussion below. 

**Table 1:** Summary of literature search and study characteristics included in this literature review AI: artificial intelligence; CXR: chest X-ray; CT: computed tomography; Sens: sensitivity; Spec: specificity; Acc: accuracy; AUC: area under the curve; CNN: convolutional neural network; CAP: community-acquired pneumonia. R-AI: Radionomics-Artificial intelligence, CO-RADS: COVID-19 Reporting and Data System, which grades CT findings from very low (1) to very high (5) suspicion for COVID-19

Study	Imaging modality	AI model	Comparator/reference	Classification task	Performance metrics	Key findings/notes
Salvatore et al., 2021 [1]	CXR	Ensemble of 10 neural networks (CNNs)	Internal hospital set (Independent Testing I) vs. multinational dataset (Independent Testing II)	COVID-19 vs. normal; COVID-19 vs. CAP	Independent testing I: Sens 98% vs. 97%, Spec 88% vs. 96%; Independent testing II: Sens 80% vs. 82%, Spec 69-89% vs. 74-84%	Model performance was highest on the internal hospital dataset and decreased with external validation. Improved accuracy in distinguishing COVID-19 pneumonia from normal CXR
Khan et al., 2024 [12]	CXR	EfficientNetB7 (transfer learning from InceptionV3, ResNet50, Xception)	Individual CNNs	COVID-19 vs. non-COVID-19 pneumonia	Acc 95% vs. 78-85%	Transfer learning improved diagnostic precision
Baltazar et al., 2021 [13]	CXR	InceptionV3, VGG, InceptionResNetV2, Xception, MobileNet	Other CNNs	3-class (Normal/COVID/non-COVID); 4-class (+bacterial/viral pneumonia)	3-class: Sens 91-96%, Spec 94-98%, PPV 90-96%; 4-class: Sens 81-86%, Spec 94-95%, PPV 81-86%	InceptionV3 demonstrated the strongest performance; accuracy declined with increasing class complexity
Enshaei et al., 2025 [14]	CXR	CV19-Net (DL model)	Four radiologists	COVID-19 vs. viral pneumonia; severity scoring	Acc 76.4% vs. 71.8%; Severity correlation 93% with radiologists	AI achieved higher accuracy and comparable severity scoring to radiologists
Zhang et al., 2021 [15]	CXR	CV19-Net	Three radiologists	COVID-19 vs. non- COVID-19 pneumonia	AUC 94% vs. 85%	AI outperformed thoracic radiologists
Li et al., 2020 [16]	CT	COVnet	—	COVID-19 vs. non-COVID-19 pneumonia	Sens 90%, Spec 96%	Deep learning model effectively extracted visual features for COVID-19 detection on CT scans
Rizzetto et al., 2022 [17]	CT	Radiomics-AI classifier	Four radiologists	CO-RADs score assignment	Radiologists: Acc 74-78%; R-AI: Acc 79%	R-AI classifier performed comparably or superior to radiologists, with greater consistency in indeterminate cases
Bai et al., 2020 [18]	CT	EfficientNetB4	Six radiologists (with vs. without AI assistance)	COVID-19 vs. non-COVID pneumonia	Acc 90% vs. 85%; Sens 88% vs. 79%; Spec 91% vs. 88%	AI assistance improved radiologists’ diagnostic accuracy, sensitivity, and specificity
Fekri-Ershad et al., 2025 [19]	CT	Dual -channel CNN	Individual texture and spatial feature channels	COVID-19 pneumonia vs. non-COVID-19	Acc 94.63%; Acc 92.74%; Acc 91.86%	Combination of both texture and spatial channels was more accurate than the channels alone
Harmon et al., 2020 [20]	CT	Hybrid 3D AI model	—	COVID-19 pneumonia vs. other lung conditions	Acc 90.8%, Sens 84%, Spec 93%	Model trained on multinational datasets to improve generalizability; external validation showed a decline
Ding et al., 2024 [21]	CXR and CT	EfficientNet;ResNet	Individual models	COVID-19 vs. normal	EfficientNet: Acc 99.44% (CXR), 99.81% (CT); Resnet: Acc 99.07% (CXR), 99.63% (CT)	EfficientNet achieved higher accuracy than ResNet across both imaging modalities

Quality Assessment

To better characterize the diagnostic performance of the included studies and evaluate their strengths and weaknesses, study quality was assessed by two independent reviewers using the QUADAS-2 tool [4]. Overall, the included studies demonstrated notable methodological limitations.

Salvatore et al. [1], Khan et al. [12], Baltazar et al. [13], Fekri-Ershad et al. [19], and Ding et al. [21] were judged to have a high risk of bias. This was primarily due to the use of retrospective and preselected datasets rather than prospective clinical cohorts or well-curated public datasets. Several studies employed image processing approaches that do not reflect real-world imaging procedures, such as extensive image augmentation or synthetic oversampling. These factors raise concern for potential overestimation of diagnostic performance.

Enshaei et al. [14], Zhang et al. [15], Li et al. [16], Rizzetto et al. [17], Bai et al. [18], and Harmon et al. [20] exhibited unclear overall risk of bias, partly due to insufficient reporting in one or more QUADAS-2 domains. Most of these studies demonstrated low risk of bias within the reference standard domain, as RT-PCR testing was used to confirm COVID-19 infection. The index test domain showed some risk of bias due to a lack of reporting on whether the AI models were interpreted independently of the reference standard. Despite this limitation, these studies generally remained applicable within the index test domain, as they addressed the diagnostic task of identifying COVID-19 pneumonia on chest imaging.

Definitive low-risk classification within the patient selection and flow and timing domains was precluded by incomplete descriptions of sampling strategies, timing between imaging and reference testing, and blinding procedures. None of the included studies achieved consistently low risk of bias across all QUADAS-2 domains.

Applicability concerns were generally consistent with the risk of bias findings. Studies that relied on retrospective and preselected datasets, highly selected cohorts, or nonclinical preprocessing methods demonstrated the greatest applicability concerns, whereas those using multinational or real-world clinical datasets showed fewer concerns [20]. Overall, these findings suggest that while AI models show promise for diagnosing COVID-19 pneumonia on imaging, methodological limitations, particularly related to study design, dataset representativeness, and reporting transparency, remain significant barriers to widespread clinical implementation. 

**Figure 4:**
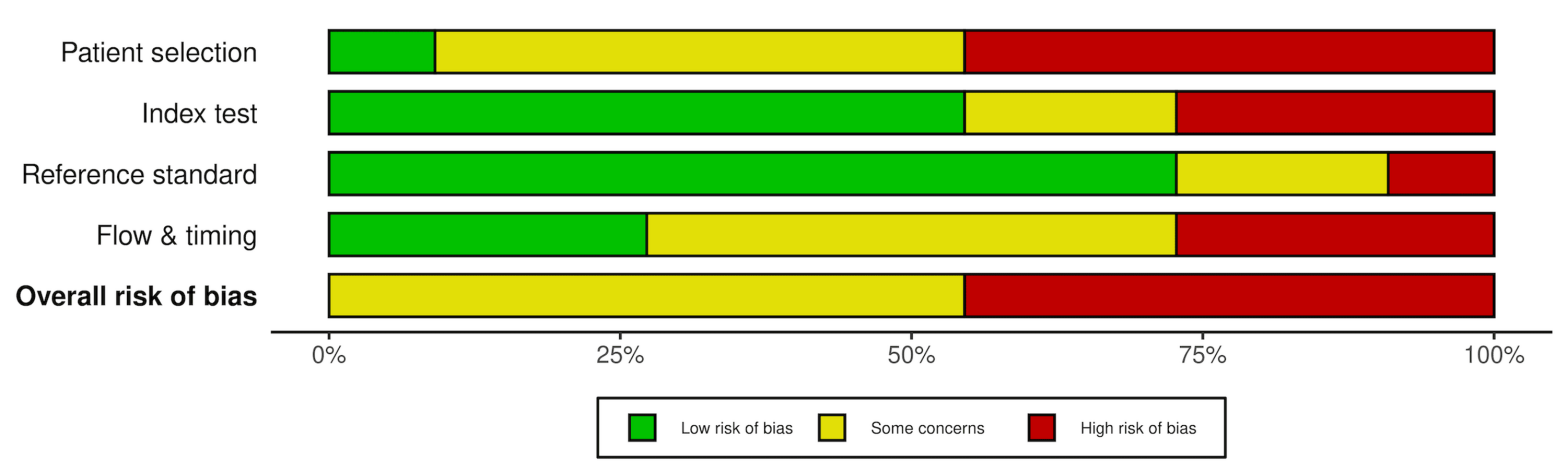
Overall and domain level risk of bias assessed using QUADAS-2 QUADAS-2: Quality Assessment of Diagnostic Accuracy Studies-2

The risk of bias and applicability concerns across included studies are summarized in Table 2 and Figure 4. Table 2 summarizes the quality assessment results of each study. Figure 4 presents a visual representation of domain-specific and overall risk of bias. 

**Table 2:** Summary of risk of bias and applicability concerns assessed using the QUADAS-2 tool QUADAS-2: Quality Assessment of Diagnostic Accuracy Studies-2

	Risk of bias				Applicability concerns		
Study	Patient selection	Index test	Reference standard	Flow and timing	Patient selection	Index test	Reference standard
Salvatore et al., 2021 [1]	high	unclear	low	low	high	low	low
Khan et al., 2024 [12]	high	low	unclear	high	high	low	moderate
Baltazar et al., 2021 [13]	high	high	low	low	high	low	low
Enshaei et al., 2025 [14]	low	unclear	low	low	low	low	low
Zhang et al., 2021 [15]	unclear	low	low	unclear	low	low	low
Li et al., 2020 [16]	unclear	low	low	unclear	moderate	moderate	low
Rizzetto et al., 2022 [17]	unclear	low	low	unclear	moderate	moderate	low
Bai et al., 2020 [18]	unclear	low	low	unclear	moderate	low	low
Fekri-Ershad et al., 2025 [19]	high	high	unclear	high	high	high	moderate
Harmon et al., 2020 [20]	unclear	low	low	unclear	low	low	low
Ding et al., 2024 [21]	high	high	high	high	high	high	high

AI Performance in COVID-19 Pneumonia CXR Detection

Recent studies have demonstrated that AI can detect COVID-19 pneumonia on CXR with promising diagnostic accuracy [1,12-15]. Salvatore et al. [1] developed an AI model comprising 10 convolutional neural networks (CNNs) to detect COVID-19 pneumonia on CXR and to distinguish it from normal findings and community-acquired pneumonia (CAP). The model was evaluated in two independent settings: internal testing within the originating hospital (Independent Testing I) and external testing utilizing a multinational dataset (Independent Testing II). The system demonstrated higher accuracy in distinguishing COVID-19 pneumonia from normal findings, particularly within the internal testing cohort [1]. Although strong performance was observed in the local dataset, diagnostic accuracy declined in the external testing set, highlighting limitations in generalizability.

Ensemble models built using transfer learning from established CNN architectures, such as EfficientNetB7, achieved higher diagnostic accuracies for COVID-19 pneumonia than their individual component networks (95% vs. 79%-86%) [12]. Baltazar et al. [13] evaluated over 9,000 CXRs using multiple CNNs, finding that InceptionV3 was the top performer, achieving 91%-96% sensitivity and 94%-98% specificity in three-class-classification models (normal, COVID-19 pneumonia, and non-COVID-19 pneumonia). Performance declined in four-class models that included bacterial and viral pneumonia-specific categories with sensitivities of 81-86% and specificities of 94-95%. 

Enshaei et al. [14] developed a deep learning (DL) model, CV19-Net, which provided consistent automated COVID-19 pneumonia severity scoring comparable to radiologist assessment. In some cases, the model demonstrated superior performance in spot diagnosis of COVID pneumonia (accuracy 76.4% vs. 71.8%). Additional studies reported that CV19-Net was able to achieve higher diagnostic accuracy than radiologists with an AUC of 94% compared with 85% [15]. Overall, AI models demonstrate strong diagnostic performance in detecting COVID-19 pneumonia on CXR, though methodological limitations remain. 

AI Performance in COVID-19 Pneumonia CT Detection

Several studies have indicated that AI models can accurately detect and classify COVID-19 pneumonia on CT imaging [16-21]. A three-dimensional (3D) DL model, COVNet, trained to extract visual features from volumetric CT scans, achieved a sensitivity of 90% and a specificity of 96% in differentiating COVID-19 pneumonia from other lung diseases [16]. 

A multireader study found that a radiomics-based AI (R-AI) classifier, which relies on quantitative imaging features rather than qualitative image assessment, performed comparably to radiologists in classifying COVID-19 pneumonia severity using the COVID-19 Reporting and Data System (CO-RADS). The R-AI achieved an overall accuracy of 79%, while radiologists demonstrated accuracies ranging from 74% in low suspicion cases to 78% in high suspicion cases [17]. 

Bai et al. [18] compared image interpretation by six radiologists with and without AI assistance and observed higher accuracy (90% vs. 85%), sensitivity (88% vs. 79%), and specificity (91% vs. 88%). Fekri-Ershad et al. [19] developed a dual-channel network that converged one AI model identifying texture features with another identifying spatial features, improving adaptability, particularly to emerging COVID-19 variants, which is an approach uncommon among prior AI models that rely on fixed feature representations. The dual-channel architecture achieved a diagnostic accuracy of 94%-95%, outperforming the individual networks.

Similarly, a study aimed to maximize generalizability of AI diagnoses through the use of a robust dataset reported that a hybrid 3D AI model combining volumetric and architectural evaluation achieved an accuracy of 90.8%, sensitivity of 84% sensitivity, and specificity of 93%. However, external validation demonstrated a decline in performance metrics [20].

Ding et al. [21] studied a DL system that combined both CXR and CT imaging analysis in the detection of COVID-19 pneumonia. EfficientNetB5 was the most accurate model evaluated, achieving accuracies of 99.4% for CXR and 99.81% for CT.

Comparison of AI with Radiologists

Several studies directly compared AI performance with that of radiologists, offering insight into the potential impact of AI in clinical workflow. Enshaei et al. [14] and Zhang et al. [15] reported that DL models outperformed experienced thoracic radiologists in distinguishing COVID-19 pneumonia from non-COVID pneumonia. Bai et al. [18] reported that radiologists' diagnostic performance metrics improved with AI assistance, highlighting the role of AI as a tool rather than a replacement for radiologists. Despite study results, AI systems remain limited in contextual reasoning, while radiologists demonstrate superior performance in managing atypical or complex cases [22].

Methodological Considerations and Challenges Unique to AI Studies

Most AI models were trained on retrospective datasets collected early in the pandemic, when disease prevalence was high, leading to class imbalance that may not reflect current clinical practice. Many studies did not report strategies to prevent data leakage, which could allow individual patients to be represented in both training and test datasets, potentially resulting in an artificially inflated accuracy [23]. Inconsistent timing between imaging and RT-PCR testing complicates interpretation due to false-negative reference standards. These methodological considerations underline the need for rigorous external validation and standardized reporting.

Limitations

Most included studies were retrospective and relied on convenience sampling, introducing selection bias. Patient selection bias represented a major limitation, which is commonly encountered in the assessment of diagnostic performance studies of emerging technologies. Study heterogeneity limited direct comparison of performance metrics. Publication bias favoring positive results, along with limited reporting of negative findings, further constrained formal synthesis and the conclusions of this review. 

Recommendations

Future research should prioritize the use of diverse, multinational datasets to enhance generalizability and reduce bias. Such datasets may improve the global applicability and diagnostic accuracy of AI models across diverse patient populations. Prospective trials and further research are necessary to further evaluate the clinical utility of AI in medical imaging settings. Consistent benchmarking against radiologist performance should be encouraged to confirm model reliability. Future studies would benefit from standardized imaging protocols and reference standards to promote uniform performance metrics. 

*Future Directions* 

AI will likely continue to expand in chest imaging beyond COVID-19 pneumonia detection toward more comprehensive pulmonary assessment, including predictions of disease severity, treatment response, and clinical outcomes. Beyond diagnostic detection, the future role of AI in COVID-19 management should focus on integrating quantitative imaging analysis with clinical and laboratory biomarkers to evaluate disease progression. Such integration would extend the role of AI beyond diagnostic detection toward comprehensive patient management [20].

## Conclusions

AI shows considerable promise in enhancing diagnostic imaging evaluation for COVID-19 pneumonia by improving accuracy, efficiency, and disease classification across radiologic modalities. While AI systems can perform comparably to human readers in controlled settings, performance often declines during external validation, underscoring the need for more diverse datasets and globally representative patient populations. Future studies should prioritize validation across varied patient populations and healthcare settings to ensure safe and generalizable clinical deployment. When appropriately utilized, AI can improve clinical workflow and provide valuable support, particularly in areas with limited radiologic resources.

Despite these advances, AI should serve as an adjunct to clinician judgment rather than replace physician diagnosis and decision-making. Future work should emphasize rigorous validation and integration with robust medical data to ensure reliable and safe clinical implementation. Strengthening AI development may improve diagnostic confidence and optimize patient outcomes across imaging disciplines beyond COVID-19 pneumonia detection. 
